# A Dual Peptide Sustained-Release System Based on Nanohydroxyapatite/Polyamide 66 Scaffold for Synergistic-Enhancing Diabetic Rats’ Fracture Healing in Osteogenesis and Angiogenesis

**DOI:** 10.3389/fbioe.2021.657699

**Published:** 2021-05-26

**Authors:** Jian Li, Jiaxing Wei, Ang Li, Hongyu Liu, Jingxue Sun, Hong Qiao

**Affiliations:** ^1^Department of Endocrinology, The Second Affiliated Hospital of Harbin Medical University, Harbin, China; ^2^Department of Orthopedics, The Second Affiliated Hospital of Harbin Medical University, Harbin, China

**Keywords:** bone tissue engineering, diabetic fracture healing, dual sustained-release, peptide, angiogenesis

## Abstract

Diabetes mellitus impairs fracture healing and function of stem cells related to bone regeneration; thus, effective bone tissue engineering therapies can intervene with those dysfunctions. Nanohydroxyapatite/polyamide 66 (n-HA/PA66) scaffold has been used in fracture healing, whereas the low bioactivity limits its further application. Herein, we developed a novel bone morphogenetic protein-2- (BMP-2) and vascular endothelial growth factor- (VEGF) derived peptides-decorated n-HA/PA66 (BVHP66) scaffold for diabetic fracture. The n-HA/PA66 scaffold was functionalized by covalent grafting of BMP-2 and VEGF peptides to construct a dual peptide sustained-release system. The structural characteristics and peptide release profiles of BVHP66 scaffold were tested by scanning electron microscopy, Fourier transform infrared spectroscopy, and fluorescence microscope. Under high glucose (HG) condition, the effect of BVHP66 scaffold on rat bone marrow mesenchymal stem cells’ (rBMSCs) adherent, proliferative, and differentiate capacities and human umbilical vein endothelial cells’ (HUVECs) proliferative and tube formation capacities was assessed. Finally, the BVHP66 scaffold was applied to fracture of diabetic rats, and its effect on osteogenesis and angiogenesis was evaluated. *In vitro*, the peptide loaded on the BVHP66 scaffold was in a sustained-release mode of 14 days. The BVHP66 scaffold significantly promoted rBMSCs’ and HUVECs’ proliferation and improved osteogenic differentiation of rBMSCs and tube formation of HUVECs in HG environment. *In vivo*, the BVHP66 scaffold enhanced osteogenesis and angiogenesis, rescuing the poor fracture healing in diabetic rats. Comparing with single peptide modification, the dual peptide-modified scaffold had a synergetic effect on bone regeneration *in vivo*. Overall, this study reported a novel BVHP66 scaffold with excellent biocompatibility and bioactive property and its application in diabetic fracture.

## Introduction

Diabetes mellitus (DM), a chronic metabolic disease common worldwide, has profound deleterious effect on fracture healing and bone formation (Jiao et al., 2015; Marin et al., 2018; Henderson et al., 2019). Diabetic patients with fractures typically show higher rates of delayed healing and non-union than non-diabetic patients, resulting in a considerable socioeconomic burden (Sundararaghavan et al., 2017; Gortler et al., 2018). Specifically, the development of microvascular complications and alterations in bone metabolism can prolong healing time by 63% (Loder, 1988). The poor bone healing is characterized by reduced bone formation, alterations in quality, composition, and biomechanical properties of bone tissue (Karim et al., 2018, 2019; Morgan et al., 2018). Previous studies demonstrated that the hyperglycemic environment decreased the population and functionality of bone marrow mesenchymal stem cells (BMSCs) and human umbilical vein endothelial cells (HUVECs), leading to a decrease in osteogenesis and angiogenesis that were key for bone regeneration (Januszyk et al., 2014; Sun et al., 2020; Xiang et al., 2020). Moreover, a low level of certain bioactive molecules that regulate BMSCs’ and HUVECs’ activity in diabetic condition might be the main reason for the above-mentioned disorders (Qiao et al., 2018; Rabbani et al., 2019; Yu et al., 2019). However, glycemic control alone does not provide satisfactory results for diabetic bone healing (Thrailkill et al., 2017). Considering the higher incidence of DM and immense healthcare-related costs generated by bone fractures, there is an urgent need to find a better strategy to efficiently augment related bioactive molecules that target diabetic-induced dysfunction of BMSCs and HUVECs to promote diabetic fracture healing (Loewenstern et al., 2019).

In bone tissue engineering, scaffolds are widely used as the matrices of bone formation (Chen et al., 2018). Based on the principle of bionics, a biomedical composite, nanohydroxyapatite/polyamide 66 (n-HA/PA66) with desirable biocompatibility and mechanical property, has been developed in recent years (Xiong et al., 2014). The n-HA/PA66 is a composite scaffold that consists of n-HA and PA66, in which the n-HA mimics the inorganic component of natural bone, and the role of PA66 is similar to collagen that is the main organic component of natural bone (Wang et al., 2007; Li et al., 2011). Besides, because of the intrinsic surface wettability and collagen-like molecular structure, it can support cellular proliferation and differentiation (Huang et al., 2020). However, the n-HA/PA66 scaffold has low bioactivity, which limits the clinical applications in fracture healing, not to mention in diabetic fracture healing.

In recent time, bone tissue engineering strategies for bioactive molecules incorporated into scaffolds have emerged and promote the stem cells’ ability to proliferate and differentiate, resulting in accelerated bone regeneration (Perez et al., 2018). Molecular signals in the form of growth factors are the main modulators of cell activity. Among them, bone morphogenetic protein-2 (BMP-2) is the most important growth factor that belongs to the transforming growth factor beta (TGF-β) super-family. BMP-2 induces bone formation *in vivo* and regulates the function of cells participating in the process of bone morphogenesis, such as BMSCs (Salazar et al., 2016). However, with the increase in the clinical use of BMP-2, side effects, such as ectopic bone formation, have emerged (James et al., 2016). Apart from BMP-2, angiogenesis-related factors are also essential for successful bone regeneration, especially in the presence of diabetes. Vascular endothelial growth factor (VEGF) is known as one of the most significant growth factors that can regulate the cell activity of HUVECs for improving vascular development and angiogenesis during osteogenesis, and it also can directly influence skeletal development (Hu and Olsen, 2016). However, in this work, gene expression of BMP-2 and vascular endothelial growth factor A (VEGFA) of BMSCs was down-regulated in high glucose (HG) condition, and in order to avoid the side effects of using growth factors, so we aimed to augment their molecular signals in a favorable form. Comparing with growth factor-based therapy, the synthetic analogs of peptide sequences from biologically relevant growth factors for remedy exhibit several advantages, including small size, low immunogenicity, and stable characteristics (Balmayor, 2015; James et al., 2016). Moreover, the short peptides with the same biological activity as growth factors can directly interact with cell receptors and stimulate particular signaling pathways (Wang et al., 2017). Consequently, how to reasonably bond peptides onto the n-HA/PA66 scaffold has aroused our interest enormously.

The traditional method is to encapsulate bioactive molecules in scaffolds *via* physical adsorption, but it could not provide sustained stimulation to cells in the long-term process of bone regeneration. To achieve a sustained-release mode, covalent bonding of peptides to scaffolds is a promising immobilization strategy that provides peptides with longer circulation half-life and improves pharmacokinetics (Maia et al., 2013; De Witte et al., 2018; Russo et al., 2020). Additionally, a dual sustained-release system acts as an attractive strategy to induce synergistic bone regeneration and improve bone healing in diabetes (Kim and Tabata, 2015; Rather et al., 2020).

**FIGURE 1:**
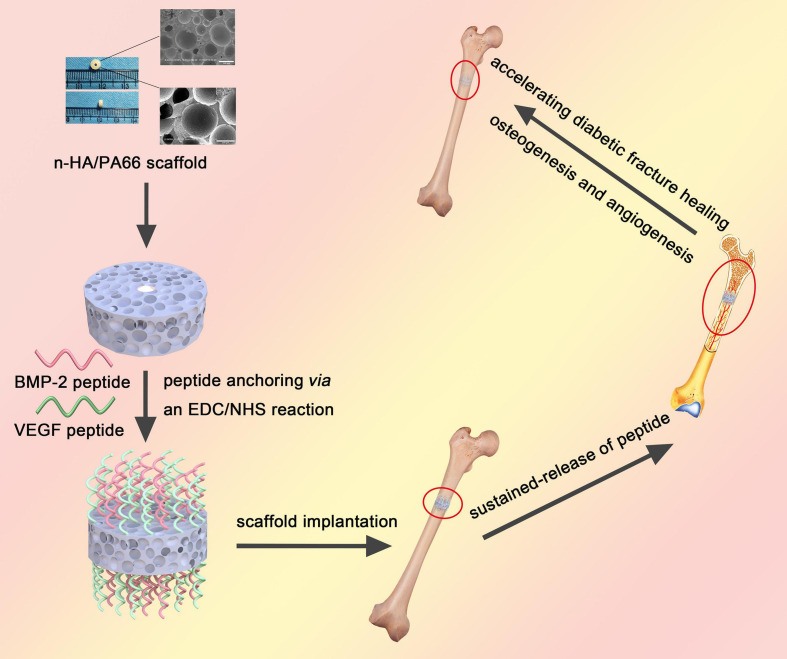
A brief schematic illustrating the synthesis of dual peptide decorated BVHP66 scaffold and its application in diabetic fracture healing.

In this work, we developed a dual sustained-release system that combined BMP-2 and VEGF peptides with the n-HA/PA66 scaffold *via* covalent bonds and that was utilized to promote osteogenesis and angiogenesis for enhancing bone regeneration in the fracture model of diabetic rats (Figure 1).

## Materials and Methods

### Materials

The n-HA/PA66 scaffolds were purchased from Sichuan Guona Technology Co., Ltd., China. BMP-2 peptides labeled with rhodamine B isothiocyanate (rhodamine B) [rhodamine B-KIPK(AC)ASSVPTELSAISTLYL sequence] and VEGF peptides labeled with fluorescein isothiocyanate (FITC) [5FITC-Acp KLTWQELYQLK(AC)YK(AC)GI sequence] were obtained from Sangon Biotech, China. N-hydroxysuccinimide (NHS), 2-(N-morpholino) ethanesulfonic acid (MES), and 1-ethyl-3-(3-dimethylaminopropyl)-carbodiimide (EDC) were purchased from Thermo Fisher Scientific, United States.

### Fabrication of Peptides Modified n-HA/PA66 Scaffolds

**FIGURE 2:**
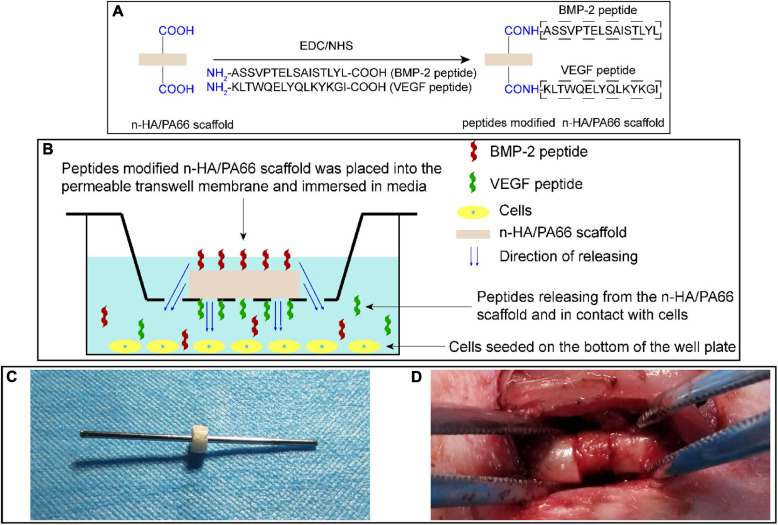
Schematic diagrams of experimental methods. **(A)** Synthetic scheme of peptide-modified n-HA/PA66 scaffold *via* an EDC/NHS reaction. **(B)** Schematic representation of cell experiments to evaluate the bioactivity of peptide-modified n-HA/PA66 scaffolds. **(C)** Images of a Kirschner wire inserted into the hollow n-HA/PA66 scaffold, and **(D)** surgical procedure of previously prepared scaffold with a Kirschner wire implanted at the fracture site of femur.

The raw n-HA/PA66 scaffold was shaped into a hollow cylinder of suitable size (outer diameter, 4.4 mm; inner diameter, 1.2 mm; height, 2.5 mm) for a Kirschner wire (diameter, 1.2 mm) inserted. BMP-2 and VEGF peptides were covalently fixed on the surface of n-HA/PA66 scaffold *via* an EDC/NHS reaction according to the manufacturer’s instructions (Figure 2A; Das et al., 2016). Briefly, the n-HA/PA66 scaffold was preactivated by incubating with a cross-linking solution of 1 ml MES, 0.4 mg EDC, and 1.1 mg NHS for 15 min. Subsequently, 1 mg BMP-2 peptides or VEGF peptides were dissolved in 1 ml phosphate-buffered saline (PBS). Afterward, the dissolved BMP-2 peptides and/or VEGF peptides were added into the cross-linking solution containing n-HA/PA66 scaffold, making the final concentration of peptides in the mixed solution 1 μg/ml, and the reaction occurred at room temperature for 2 h. Finally, all the scaffolds were washed with PBS three times to remove the unbound peptides. Scaffolds modified with different peptides were divided into the following groups: (1) HP66 scaffold, the n-HA/PA66 scaffold without loading any peptide; (2) BHP66 scaffold, the n-HA/PA66 scaffold modified with BMP-2 peptides; (3) VHP66 scaffold, the n-HA/PA66 scaffold modified with VEGF peptides; and (4) BVHP66 scaffold, the n-HA/PA66 scaffold modified with BMP-2 peptides and VEGF peptides.

### Characteristics of Scaffolds

The macroscopic images of n-HA/PA66 scaffold were taken, and the microscopic morphology of n-HA/PA66 scaffold was captured by scanning electron microscopy (SEM, S-3400n; Hitachi, Japan). Additionally, the n-HA/PA66 scaffold’s compressive strength was determined using an electronic universal testing machine (AGXplus; SHIMADZU, Japan).

Fourier transform infrared spectroscopy (FT-IR, Nicolet is50; Thermo Fisher Scientific, United States) was used to identify amide bonds formation through covalent cross-linking between the n-HA/PA66 scaffolds’ carboxyl bonds and peptides’ amino bonds (Shen et al., 2009). Furthermore, we observed the cross-linking of peptides and n-HA/PA66 scaffold by fluorescence microscopy with an exposure time of 100 ms (DMI4000B; Leica, Germany).

### Peptide Release Profiles of BVHP66 Scaffold

The modified scaffold was incubated with 200 μl PBS for peptide release, and 100 μl supernatant was collected on 1, 2, 3, 4, 5, 6, 7, 10, and 14 days and then mixed with additional 100 μl PBS. The fluorescence intensity of rhodamine B-labeled BMP-2 peptides (with excitation and emission wavelengths of 555 and 580 nm) and FITC-labeled VEGF peptides (with excitation and emission wavelengths of 490 and 525 nm) in the acquired solution was detected by a microplate reader (Infinite M200; Tecan, Switzerland) to obtain the peptide cumulative release (Weng et al., 2018). Moreover, the initial mass of peptides loaded on the scaffold was calculated from the total mass of peptides in the cross-linking solution, minus the mass of peptides discarded by washing the scaffold and remained in the cross-linking solution. Furthermore, the peptide cumulative release profiles were analyzed.

### Isolation, Culture, and Identification of Rats’ BMSCs

Three-week-old Sprague–Dawley (SD) rats were purchased from the Animal Experiment Center of the Second Affiliated Hospital of Harbin Medical University. All the experiments were approved by the ethical committee of Harbin Medical University (approval number, SYDW2019-2). The rats were euthanized by CO_2_ inhalation, and the rat bone marrow mesenchymal stem cells (rBMSCs) were isolated by the standard method of whole bone marrow adherence as described (Fang et al., 2019).

For HG condition, D-glucose was added to the medium to mimic the hyperglycemic microenvironment *in vitro*. Then, the rBMSCs were cultured in minimum essential medium (MEM; Gibco, United States) with HG or normal glucose (CON) (glucose concentrations 30 or 5.5 mmol/L, respectively) supplemented with 10% fetal bovine serum (FBS; Diagnovum, Germany), 1% penicillin/streptomycin, 1% non-essential amino acids (NEAA; Gibco, United States), 10 ng/ml fibroblast growth factor basic (bFGF; PeproTech, United States), and 10 ng/ml epidermal growth factor (EGF; PeproTech, United States) at 37°C and 5% CO_2_ conditions. The rBMSCs at passage 3 were used for the *in vitro* experiments.

To identify the isolated cells, passage 3 cells were stained with the following antibodies: CD29-APC, CD90-PE, CD11b/c-PE-Cy7, and CD45-FITC (BioLegend, United States). Then, the analyses were conducted on a flow cytometer (novocyte 3110; ACEA, United States).

### Adhesion and Proliferation of rBMSCs Seeded on the Scaffolds

To evaluate the cytocompatibility, the images of rBMSCs adhered to the surface of BVHP66 scaffold were captured by SEM on 7 and 14 days. Additionally, the proliferation capacities of rBMSCs seeded on different scaffolds were quantified using a Cell Counting Kit-8 (CCK-8) assay. Specifically, scaffolds were pre-immersed in an osteogenic-defined medium (Cyagen, United States) overnight. Then, the rBMSCs (5 × 10^4^ cells) in 1 ml osteogenic-defined medium were seeded on each scaffold in each well of 24-well plates, and the plates were incubated at 37°C and 5% CO_2_ conditions. On 1, 3, 5, 7, 10, and 14 days, the CCK-8 solution (Meilunbio, China) was added to each well, and then they were incubated for 4 h. The absorbance values were evaluated at 450 nm using a microplate reader (Multiskan FC; Thermo Fisher Scientific, United States). The rBMSCs were divided into five groups based on cultured in different glucose concentrations of medium and seeded with different peptide-modified scaffolds: CON + HP66 group, HG + HP66 group, HG + BHP66 group, HG + VHP66 group, and HG + BVHP66 group.

### Alkaline Phosphatase Staining and ALP Activity Measurement

In order to determine the effects of different scaffolds on osteogenic differentiation of rBMSCs, we carried out the following experiment. For osteogenic induction, the culture medium was changed to the osteogenic-defined medium. Furthermore, rBMSCs cultured in a non-osteogenic medium (non-OM group) served as a control. The cells were seeded on the bottom of 24-well plates and cultured in the medium. Each scaffold was placed in each transwell insert on the permeable membrane (Corning, United States); thus, BMP-2 and VEGF peptides were released from the scaffold, filtered through the membrane, and allowed to contact the cells (Figure 2B).

Alkaline phosphatase (ALP) staining was used to evaluate the early osteogenesis capacities of rBMSCs in different groups. For ALP staining, rBMSCs were washed with PBS and fixed with 4% paraformaldehyde for 10 min on day 7 after osteoinduction. Next, the cells were incubated with BCIP/NBT ALP Color Development Substrate (Beyotime, China) for 30 min, following the manufacturer’s instruction. Then, each well was imaged after additional washing. Afterward, the ALP activity was assessed by an ALP assay kit (Nanjing Jiancheng, China) after breaking up the cells by ultrasound. The optical density (OD) values at 520 nm were measured using a microplate reader (Infinite M200; Tecan, Switzerland).

### Alizarin Red S Staining and Quantitative Mineralization Assay

The Alizarin Red S (ARS) staining was used to assess the effects of different scaffolds on calcium mineralization of rBMSCs in the final stage of osteogenesis (Figure 2B). The procedure of ARS staining was described as follows. The cells were washed twice with PBS and fixed with 4% paraformaldehyde for 10 min on day 14 after osteoinduction. Next, the cells were incubated with ARS solution for 10 min at room temperature (Cyagen Biosciences, China) according to the manufacturer’s instruction. Then, the ARS solution was discarded, and each well was imaged after additional washing. Subsequently, the calcium mineralization was quantified by dissolving ARS with cetylpyridinium chloride (Sigma-Aldrich, United States). Then, the supernatant solution of each well was transferred to a 96-well plate, and a microplate reader (Multiskan FC; Thermo Fisher Scientific, United States) was used to measure the OD values at 560 nm.

### RNA Extraction and Quantitative Real-Time PCR

To compare the mRNA expressions of osteogenic and angiogenic genes of rBMSCs in the medium with HG and normal glucose and to further evaluate the effects of different scaffolds on osteogenic differentiation capacity of rBMSCs, the mRNA expressions of collagen type I alpha 1 (Col1a1) on day 7 after osteoinduction were determined, whereas BMP-2, VEGFA, runt-related transcription factor 2 (Runx2), osteocalcin (OCN), and osteopontin (OPN) on day 14 after osteoinduction were determined (Figure 2B). The total RNA was extracted from the cells with TRIzol Reagent (Thermo Fisher Scientific, United States) and reverse transcribed to cDNA using the ReverTra Ace qPCR RT Kit (TaKaRa, Japan). Then, the quantitative real-time PCR (qPCR) was carried out using TB Green Premix Ex Taq II (TaKaRa, Japan) as described by the manufacturer. The primers were shown in Table 1, and the relative gene expression was calculated using the 2^−ΔΔCt^ method.

**TABLE 1:** PCR primer sequences.

cDNA	Primer	Sequences
BMP-2	Forward	5′-TGTGAGGATTAGCAGGTCTTTG-3′
	Reverse	5′-TTGTGGAGTGGATGTCCTTTAC-3′
VEGFA	Forward	5′-GGATCAAACCTCACCAAAGCCA-3′
	Reverse	5′-TTGGTCTGCATTCACATCTGCT-3′
Runx2	Forward	5′-TGGCCTTCCTCTCTCAGTAA-3′
	Reverse	5′-GTAAGTGAAGGTGGCTGGATAG-3′
Col1a1	Forward Reverse	5′-ACTGGTACATCAGCCCAAAC-3′ 5′-GGAACCTTCGCTTCCATACTC-3′
OPN	Forward	5′-TGAGTTTGGCAGCTCAGAGGAGAA-3′
	Reverse	5′-ATCATCGTCCATGTGGTCATGGCT-3′
OCN	Forward Reverse	5′-CTGAGTCTGACAAAGCCTTCA-3′ 5′-TCCAAGTCCATTGTTGAGGTAG-3′

### Culture and Proliferation of HUVECs

HUVECs (Cell Bank of the Chinese Academy of Sciences, China) were grown to confluence in endothelial cell medium (ECM; Sciencell, United States) at 37°C in a cell incubator under 5% CO_2_. The HUVECs were maintained in physiological glucose (CON, 5.5 mmol/L) or HG (30 mmol/L) concentrations.

To evaluate the cell viability of HUVECs, the cells (3 × 10^4^ cells/well) were seeded on the bottom of 24-well plates, and each scaffold was placed in each upper chamber of transwells (Figure 2B). Then, the cells were cultured in ECM for 24, 48, 72, and 96 h at 37°C. The cells were exposed to BMP-2 peptide and/or VEGF peptide that were released from n-HA/PA66 scaffold. The CCK-8 solution was added to each well of the plates, and then they were incubated for 4 h. Next, the OD values were measured at 450 nm by a microplate reader (Multiskan FC; Thermo Fisher Scientific, United States). HUVECs were divided into five groups based on cultured in varied glucose concentrations of medium and treated with different peptide-decorated scaffolds: CON + HP66 group, HG + HP66 group, HG + BHP66 group, HG + VHP66 group, and HG + BVHP66 group.

### Tube Formation Assay of HUVECs

The effect of different scaffolds on angiogenesis of HUVECs was assessed by tube formation assay. In brief, 10 μl Matrigel (BD Biosciences, United States) was placed into each well of the precooled ibiTreat (Ibidi, Germany) on ice and incubated at 37°C for 30 min, and then HUVECs (3 × 10^4^ cells/well) were seeded on it. Subsequently, 10 μl supernatant containing peptides released from the scaffold of different groups in PBS on day 14 was added to each well. After 4 h of incubation at 37°C, the images of tube formation were captured using a microscope (Olympus, Japan) and analyzed by ImageJ software 1.52a (Rawak Software Inc., Germany).

### Animals and Induction of Type 1 Diabetes Mellitus

The 8-week-old male SD rats weighting 230–250 g were purchased from the Animal Experiment Center of the Second Affiliated Hospital of Harbin Medical University. Type 1 diabetes mellitus (T1DM) was induced by a single intraperitoneal injection of streptozotocin (STZ, 60 mg/kg; Sigma-Aldrich, United States) dissolved in 0.1 mmol/L sodium citrate buffer (Solarbio, China) at pH 4.4. After 7 days, caudal vein blood was collected to detect blood glucose by a contour glucose meter (Roche, Germany). Rats were considered diabetic when blood glucose levels exceeded 16.7 mmol/L.

### Femoral Fractures

At 3 weeks following stable DM condition, the rats were placed under general anesthesia. The right mid-femur (diameter, 4.4 mm) of each rat was osteotomized transversely by a wire saw. The previously prepared scaffold was implanted at the fracture site, and then a Kirschner wire went through the hole of scaffold and came out from both ends of the femur to achieve an intramedullary fixation (Figures 2C,D). Finally, an intramuscular injection of penicillin was administered postoperatively to prevent infection. At 4 or 8 weeks after fractures, rats were sacrificed, and radiological and histological analyses were performed. Forty-eight rats were divided randomly into the following six groups: non-DM group, non-diabetic rat without scaffold implanted; DM group, diabetic rat without scaffold implanted; DM + HP66 group, diabetic rat with n-HA/PA66 scaffold implanted; DM + BHP66 group, diabetic rat with BHP66 scaffold implanted; DM + VHP66 group, diabetic rat with VHP66 scaffold; and DM + BVHP66 group, diabetic rat with BVHP66 scaffold implanted.

### X-Ray and Micro-CT Examinations

The right femora were excised at 4 and 8 weeks post-surgery and then fixed in 4% paraformaldehyde. Radiographs were obtained in all six groups by an X-ray machine (FAXITRON, MC20, United States). The radiographic healing score of radiographs was assessed independently by three observers (grade 1, no calcification; grade 2, patchy calcification; grade 3, calcification with the appearance of a callus; grade 4, callus bridging the fracture gap; grade 5, continuity of the bone trabecula; and grade 6, bone remodeling) (Quirk et al., 2016). After removing Kirschner wires, the femora in the DM + HP66, DM + BHP66, DM + VHP66, and DM + BVHP66 groups were scanned using a micro-CT system (Rigaku, Japan) at a resolution of 20 μm with the following parameters: current, 88 μA and X-ray energy, 90 kVp. The region of interest was 200 axial slices above and below the fracture line, and then bone volume fraction (BV/TV), connectivity density (Conn.D), trabecular thickness (Tb.Th), and trabecular spacing (Tb.Sp) were calculated by ImageJ software 1.52a.

### Histological Examination

The femora were decalcified for 8 weeks in ethylenediaminetetraacetic acid (EDTA) solution at room temperature. Afterward, the specimens were dehydrated with a series of ethanol rinses and then embedded in paraffin. By a microtome (Leica, Germany), 5 μm thick sections were obtained along the femora’s sagittal plane. H&E staining and Masson’s trichrome staining were performed following the manufacturer’s instructions. Immunohistochemical (IHC) staining for CD31 (Abcam, United Kingdom) was performed on paraffin sections to detect neovascularization. Finally, measurements of vascular density at the fracture site were performed.

### Statistical Analysis

Statistical analysis was performed using SPSS 21.0 (SPSS Inc., United States) and GraphPad Prism 6.0 (GraphPad Software Inc., United States). Data were expressed as the mean ± standard deviation, each *n* = 3. The differences between two groups were compared with *t*-test. One-way analysis of variance (ANOVA) was used to assess the statistical significance of at least three separate trials. In all values, *P* < 0.05 was considered significant in the experiment.

## Results

### The Structural and Mechanical Characteristics of n-HA/PA66 Scaffold

**FIGURE 3:**
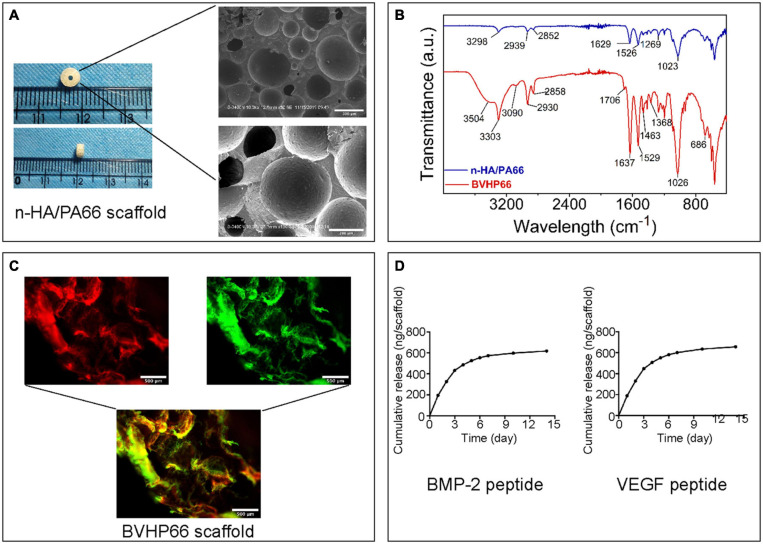
Characteristics of n-HA/PA66 scaffold and BVHP66 scaffold. **(A)** Macrographs and SEM micrographs of shaped n-HA/PA66 scaffold. **(B)** FT-IR spectra of n-HA/PA66 scaffold and BVHP66 scaffold. **(C)** Fluorescent microscope images of rhodamine B-BMP-2 peptide and FITC-VEGF peptide-modified n-HA/PA66 scaffold. **(D)** *In vitro* cumulative release profiles of BMP-2 and VEGF peptides from BVHP66 scaffold. Scale bar = 500 and 200 μm. *n* = 3 for each group.

The n-HA/PA66 scaffold was shaped as a hollow cylinder, and its outer and inner diameters were, respectively, consistent with the diameters of the mid-femur and the Kirschner wire. SEM images illustrated that the n-HA/PA66 scaffold displayed highly interconnecting pores with various sizes (Figure 3A). Additionally, the mechanical test was performed on the shaped n-HA/PA66 scaffold, and the compressive strength was 14.88 ± 0.72 MPa (*n* = 3).

### Characteristics of BVHP66 Scaffold

FT-IR was used to determine the chemical modification of BVHP66 scaffold after cross-linking reaction. Figure 3B shows the FT-IR spectra of pure n-HA/PA66 scaffold (blue spectra) and the dual peptides modified n-HA/PA66 scaffold (red spectra). The main functional groups of PA66 are amide I and C–H. As shown by the blue spectra in Figure 3B, the peak at 1,629 cm^–1^ corresponded to amide I, the peak at 1,023 cm^–1^ corresponded to C–O, and the peaks at 2,852 and 2,939 cm^–1^ could be assigned to C–H. When modified with the peptides, as shown by the red spectra in Figure 3B, the 3,303 cm^–1^ absorption could be assigned to –NH, whereas the peak at 3,504 cm^–1^ could be assigned to N–H vibrations that were also found in PA66. Moreover, the 1,706 cm^–1^ peak could be related to C = O in carboxylic acids. These results indicated the successful decorating of BMP-2 and VEGF peptides by the amide covalent bonds between n-HA/PA66 scaffold and peptides (Wen et al., 2019).

To investigate peptides’ binding and releasing on the n-HA/PA66 scaffold’s surface, rhodamine B-labeled BMP-2 peptide and FITC-labeled VEGF peptide were utilized. As exhibited in Figure 3C, the vivid fluorescence was observed on the BVHP66 surface. The red fluorescence ascribes to rhodamine B-BMP-2 peptide, and the green fluorescence stems from FITC-VEGF peptide, whereas the merged image of the above two displays a bright yellow fluorescence, implying the successful functionalization of the dual peptides on the BVHP66 surface.

### Peptide Release Profiles of BVHP66 Scaffold

The *in vitro* cumulative release profiles of BMP-2 and VEGF peptides from n-HA/PA66 scaffold for 14 days were assessed. It can be seen from Figure 3D that about 433.65 ng BMP-2 peptides and 447.47 ng VEGF peptides were released within 3 days, indicating that 51.86% BMP-2 peptides and 52.83% VEGF peptides were initially released, respectively. Then, the peptide releasing reached a plateau and kept in a slow-sustained mode. Approximately 619.06 ng BMP-2 peptides and 657.80 ng VEGF peptides were released in total over 14 days, with a cumulative release percentage of about 74.04 and 77.66%, respectively. The results above suggest that the release of these two peptides consists of two stages. One is the early burst-release. The other is the slow-sustained release in the following stage.

### Identification of rBMSCs

**FIGURE 4:**
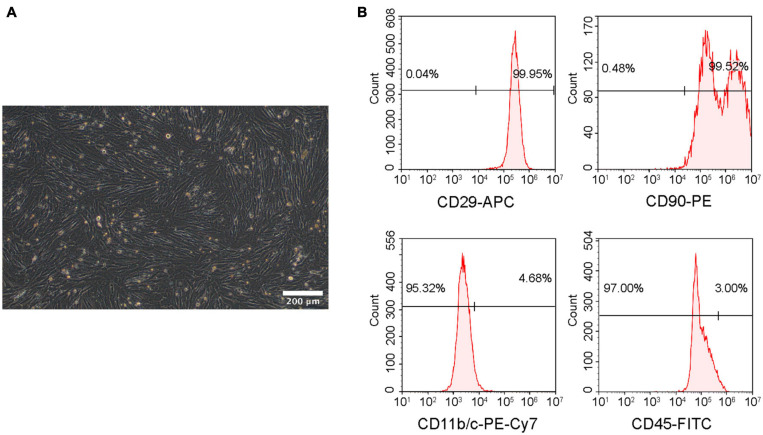
Identification of rBMSCs. **(A)** Microscopic image of rBMSCs. Scale bar = 200 μm. **(B)** Surface marker analysis of rBMSCs.

The isolated cells were identified from morphology and surface characteristic markers using microscopy and flow cytometry. Passage 3 rBMSCs exhibited a fibroblast-like or long fusiform shape morphology (Figure 4A). The analysis of flow cytometry demonstrated that the cells were positive for CD29 (99.95%) and CD90 (99.52%) and negative for CD45 (3.00%) and CD11b/c (4.68%) (Figure 4B), which demonstrated that the isolated cells were rBMSCs.

### Adhesion and Proliferation of rBMSCs

**FIGURE 5:**
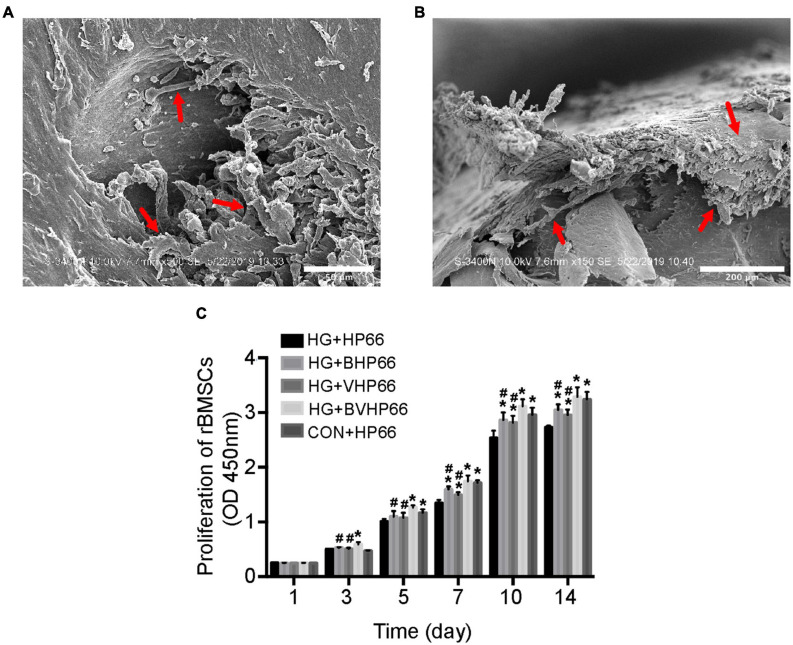
Adhesion and proliferation of rBMSCs. SEM micrographs of rBMSCs adhered to the surface of BVHP66 scaffold after cultured for **(A)** 7 and **(B)** 14 days. **(C)** The proliferation of rBMSCs in different groups on different days. Scale bar = 50 and 200 μm. *n* = 3 for each group, data were expressed as mean ± SD. *P < 0.05, vs. HG + HP66 group; # *P* < 0.05, vs. HG + BVHP66 group.

The SEM images revealed that well-stretched rBMSCs could adhere to the surface of BVHP66 scaffold and connect to each other after 7 and 14 days (Figures 5A,B). As Figure 5C depicted, the number of rBMSCs increased with time in all the groups. The number of rBMSCs between the CON + HP66 and HG + HP66 groups indicated no significant difference on 1 and 3 days, whereas the number of rBMSCs in the CON + HP66 group was more than that in the HG + HP66 group on 5, 7, 10, and 14 days (*P* < 0.05). On 3 and 5 days, there was no noticeable difference in cell viability of rBMSCs among the HG + HP66, HG + BHP66, and HG + VHP66 groups, whereas cell viability of rBMSCs in the HG + BVHP66 group presented significantly greater than that in the HG + HP66 group (*P* < 0.05). Besides, the number of rBMSCs in the HG + BHP66, HG + VHP66, and HG + BVHP66 groups was more than that in the HG + HP66 group, and the number of rBMSCs in the HG + BVHP66 group was more than that in the HG + BHP66 and HG + VHP66 groups on 7, 10, and 14 days (*P* < 0.05). Additionally, the number of rBMSCs was similar between the HG + BVHP66 group and the CON + HP66 group at every point of time.

### Osteogenic Differentiation of rBMSCs

**FIGURE 6:**
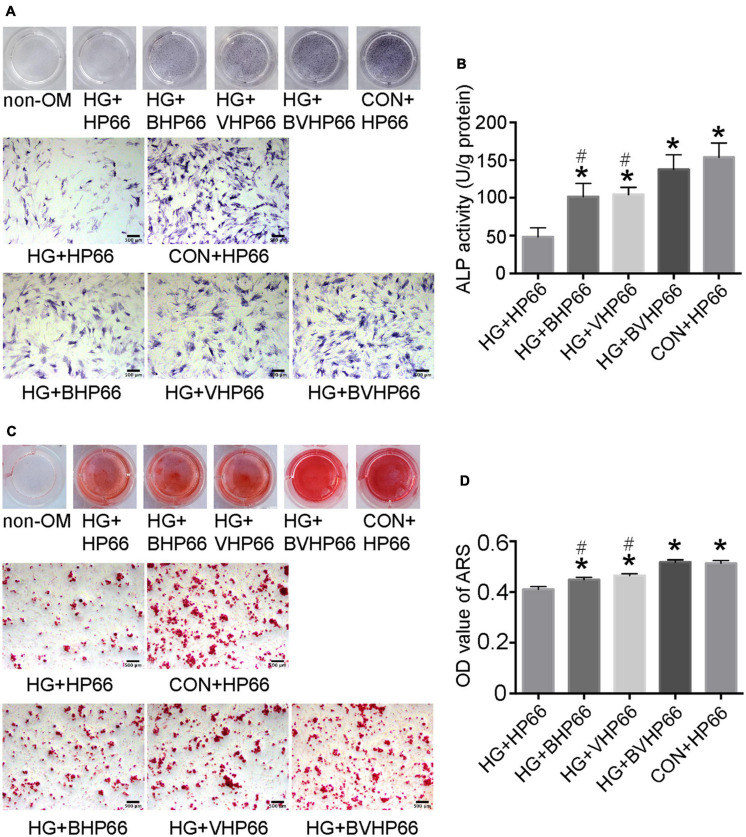
Osteogenic differentiation of rBMSCs. **(A)** ALP staining and **(B)** ALP activity were evaluated on day 7 after cultured in osteogenic medium. **(C)** The ARS staining and **(D)** the quantitative mineralization assay were conducted on day 14 after cultured in osteogenic medium. Scale bar = 500 μm. *n* = 3 for each group, data were expressed as mean ± SD. *P < 0.05, vs. HG + HP66 group; # *P* < 0.05, vs. HG + BVHP66 group.

To investigate the effect of BVHP66 scaffold on osteogenic differentiation of rBMSCs, ALP staining, ARS staining, and qPCR experiments were conducted. On day 7, the ALP-positive area in the HG + HP66 group was significantly smaller than that in the CON + HP66 group (Figure 6A). The osteogenic-induced rBMSCs from the HG + BHP66, HG + VHP66, and HG + BVHP66 groups showed more positive ALP staining than those from the HG + HP66 group. The HG + BVHP66 group exhibited the most robust positive ALP staining in HG condition. Moreover, the semiquantitative analysis of ALP activity showed a similar trend (*P* < 0.05, Figure 6B). As shown by ARS staining and the quantitative mineralization analysis, the CON + HP66 group displayed a larger amount of calcium deposition than the HG + HP66 group on day 14 (*P* < 0.05, Figures 6C,D). Additionally, it further revealed that the HG + BVHP66, HG + BHP66, and HG + VHP66 groups showed significantly larger calcium deposition than the HG + HP66 group, whereas the HG + BVHP66 group exhibited greater calcium deposition than the HG + BHP66 and HG + VHP66 groups (*P* < 0.05). Furthermore, our ALP staining and ARS staining results showed that the ability of osteogenic differentiation of rBMSCs between the HG + BVHP66 group and the CON + HP66 group showed no significant difference (Figure 6).

**FIGURE 7:**
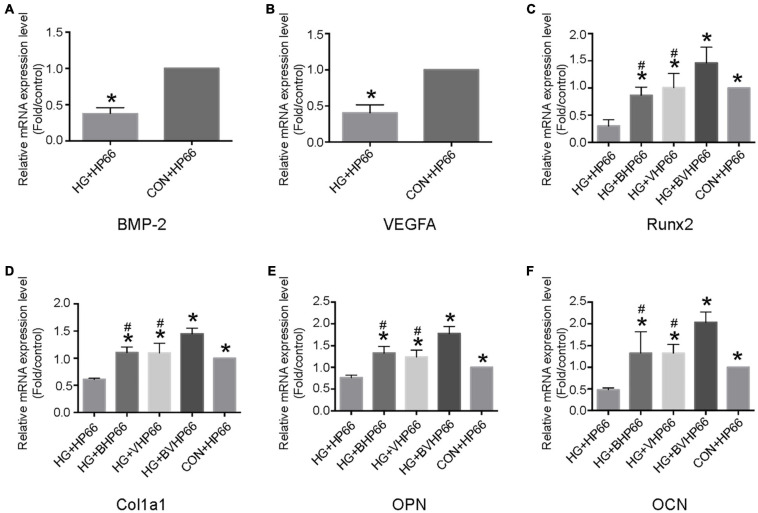
Osteogenesis- and angiogenesis-related gene expressions of rBMSCs. Relative mRNA expression levels of **(A)** BMP-2 and **(B)** VEGFA in the HG + HP66 and CON + HP66 groups, **(C)** Runx2, **(D)** Cola1, **(E)** OPN, and **(F)** OCN in the HG + HP66, HG + BHP66, HG + VHP66, HG + BVHP66, and CON + HP66 groups. *n* = 3 for each group, data were expressed as mean ± SD. *P < 0.05, vs. HG + HP66 group; # *P* < 0.05, vs. HG + BVHP66 group.

The osteogenic differentiation of rBMSCs is bound to be accompanied by a cascade of intracellular regulation of gene expression. As illustrated in Figure 7, BMP-2, VEGF, Runx2, Cola1, OPN, and OCN showed lower gene expression levels in the HG + HP66 group than in the CON + HP66 group (*P* < 0.05). The Runx2, Cola1, OPN, and OCN displayed higher gene expression levels in the HG + BHP66, HG + VHP66, and HG + BVHP66 groups than in the HG + HP66 group (*P* < 0.05). Meanwhile, the HG + BVHP66 group revealed the highest gene expression levels in HG condition (*P* < 0.05). Moreover, gene expression levels in the HG + BVHP66 group were not significantly lower than those in the CON + HP66 group. The above results indicated that the BMP-2 and VEGF peptides released from n-HA/PA66 scaffold promoted the osteogenic differentiation of rBMSCs and the combination of the two was the most effective.

### Proliferation and Tube Formation of HUVECs

**FIGURE 8:**
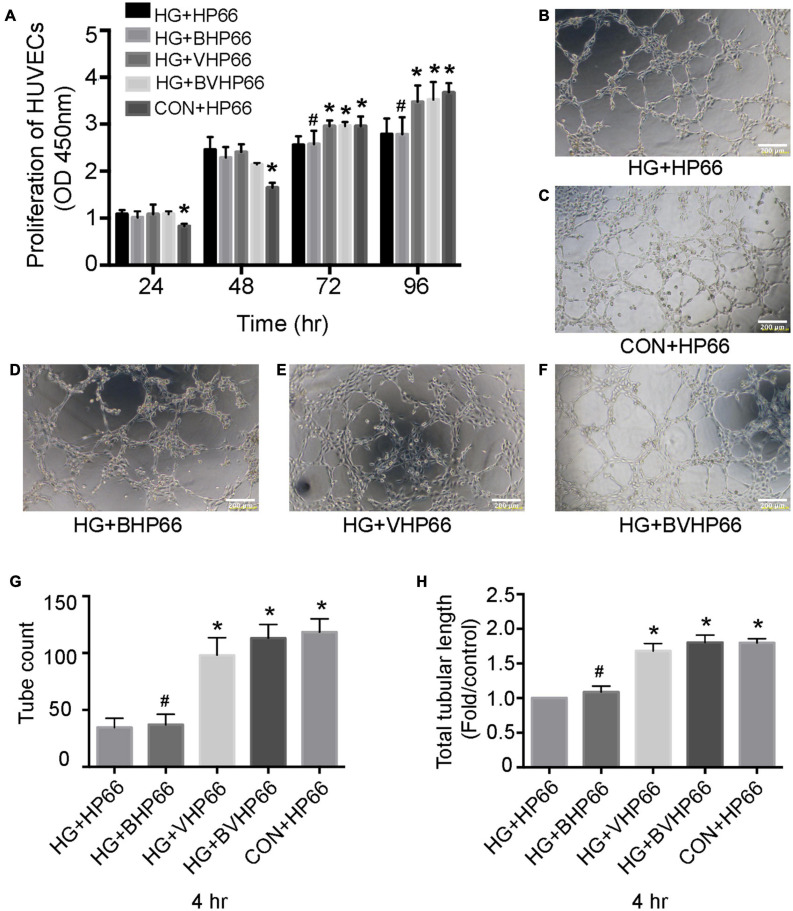
Proliferation and tube formation of HUVECs. **(A)** The proliferation of HUVECs in different groups at different hours. **(B–F)** Representative images of tube formation after incubated for 4 h. The results of **(G)** tube count and **(H)** total tubular length. Scale bar = 200 μm. *n* = 3 for each group, data were expressed as mean ± SD. *P < 0.05, vs. HG + HP66 group; # *P* < 0.05, vs. HG + BVHP66 group.

As shown in Figure 8A, HUVECs in the HG + HP66 group showed a greater proliferation capacity than those in the CON + HP66 group in the first 24 and 48 h, whereas the capacity of HUVECs proliferation in the HG + HP66 group was poorer than that in the CON + HP66 group at 72 and 96 h (*P* < 0.05). The proliferative capacity of HUVECs in the HG + BHP66, HG + VHP66, and HG + BVHP66 groups revealed no apparent difference compared with the HG + HP66 group at 24 and 48 h. Then, HUVECs exhibited higher cell viability in the HG + VHP66 and HG + BVHP66 groups than in the HG + HP66 and HG + BHP66 groups (*P* < 0.05), whereas the cell viability exhibited no significant difference among the HG + VHP66 group, HG + BVHP66 group, and CON + HP66 group, and that also showed no evident significance between the HG + HP66 group and HG + BHP66 group at 72 and 96 h.

Moreover, according to the Matrigel-based capillary genesis assay, the tube formation capacity of HUVECs was poorer in the HG + HP66 group than in the CON + HP66 group (Figures 8B,C). Additionally, tube formation was evidently promoted in the HG + VHP66 and HG + BVHP66 groups compared with the HG + HP66 and HG + BHP66 groups (Figures 8B,D–F). The tube formation capacity of HUVECs showed no significant difference between the HG + HP66 group and the HG + BHP66 group (Figures 8B,D). Moreover, there was no evident difference in tube formation capacity of HUVECs among the CON + HP66, HG + VHP66, and HG + BVHP66 groups (Figures 8C,E,F). Quantitatively, the tube count (Figure 8G) and total tubular length (Figure 8H) were decreased in the HG + HP66 group compared with the CON + HP66 group (*P* < 0.05). These parameters were enhanced in the HG + VHP66 and HG + BVHP66 groups compared with the HG + HP66 and HG + BHP66 groups (*P* < 0.05). The parameters presented no obvious differences among the CON + HP66, HG + VHP66, and HG + BVHP66 groups, and those also exhibited no significant difference between the HG + HP66 group and the HG + BHP66 group.

### X-Ray and Micro-CT Analyses

**FIGURE 9:**
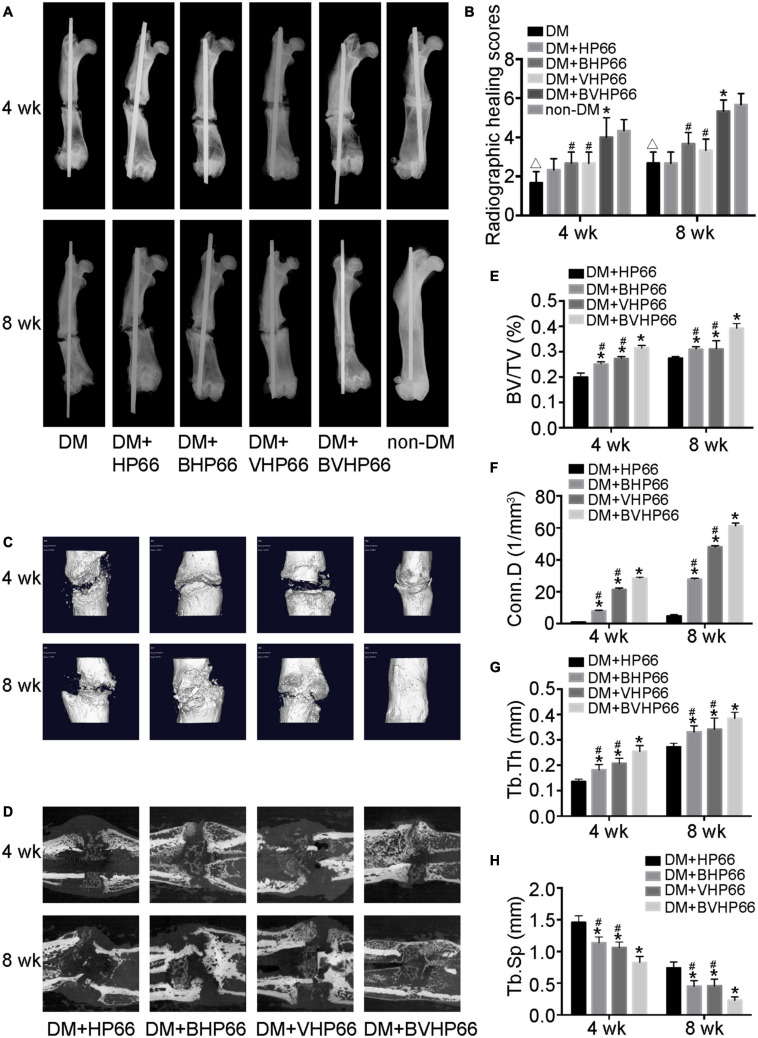
X-ray and micro-CT analyses. **(A)** Representative radiographs and **(B)** radiographic healing scores of all the groups after 4 and 8 weeks. Representative **(C)** 3D and **(D)** 2D micro-CT images around the fracture site after 4 and 8 weeks in the DM + HP66, DM + BHP66, DM + VHP66, and DM + BVHP66 groups. The quantitative analysis of regenerated bone structure, including **(E)** BV/TV, **(F)** Conn.D, **(G)** Tb.Th, and **(H)** Tb.Sp. *n* = 3 for each group, data were expressed as mean ± SD. *P < 0.05, vs. HG + HP66 group; # *P* < 0.05, vs. HG + BVHP66 group; △*P* < 0.05, vs. non-DM group.

The effect of BVHP66 scaffold on diabetic bone regeneration was evaluated in the fracture model of diabetic rats. Four and 8 weeks after surgery, X-ray and micro-CT were performed to evaluate new bone formation around the fracture site. Compared with the non-DM group, the DM group had worse fracture healing and lower radiographic healing score, as proven by X-ray analysis (*P* < 0.05, Figures 9A,B). Moreover, two-dimensional (2D) and three-dimensional (3D) micro-CT images (sagittal and frontal views) suggested that the DM + BHP66, DM + VHP66, and DM + BVHP66 groups exhibited more bone formation than the DM + HP66 group (Figures 9C,D). Additionally, unlike other groups, diabetic rat implanted with BVHP66 scaffold and non-diabetic rat without implantation displayed complete fracture healing in the eighth week (Figures 9A–D). Compared with the DM + HP66 group, the DM + BHP66, DM + VHP66, and DM + BVHP66 groups manifested structural changes in new bone by the quantitative analysis of the micro-CT scans, including an increase in BV/TV, Conn.D, and Tb.Th but a decrease in Tb.Sp (*P* < 0.05, Figures 9E–H). Among them, diabetic rat implanted with BVHP66 scaffold exhibited the most obvious changes in bone structure parameters in the eighth week (*P* < 0.05, Figures 9E–H). In the X-ray and micro-CT assessments, the BVHP66 scaffolds revealed an excellent capacity in enhancing bone regeneration, which surpassed single peptide-modified scaffolds (*P* < 0.05, Figures 9B, E–H).

### Histological Analysis

**FIGURE 10:**
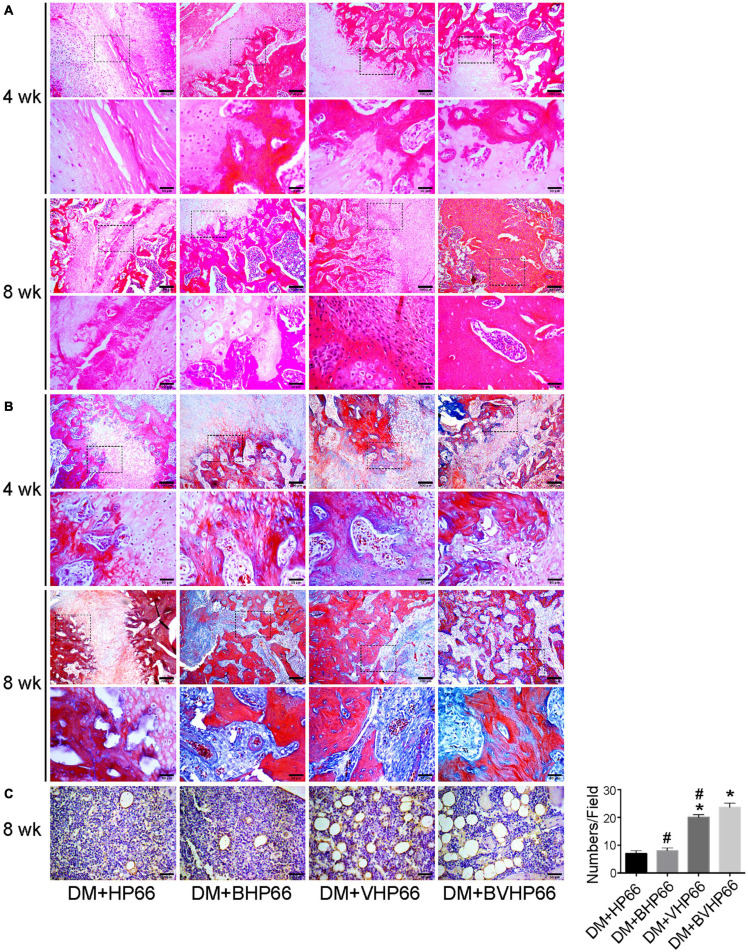
Histological analysis. Representative histological sections after **(A)** H&E staining, **(B)** Masson’s trichrome staining, and **(C)** immunohistochemistry of CD31. Moreover, the number of vessels in the field was displayed in the graph. Scale bar = 200 and 50 μm. *n* = 3 for each group, data were expressed as mean ± SD. *P < 0.05, vs. HG + HP66 group; # *P* < 0.05, vs. HG + BVHP66 group.

H&E and Masson’s trichrome images validated the results of X-ray and micro-CT experiments. In the fourth week, the results of H&E staining showed smaller and thinner new bone tissue and more extensive fibrous tissue in the DM + HP66 group than in the DM + BHP66, DM + VHP66, and DM + BVHP66 groups (Figure 10A). Moreover, in the DM + BVHP66 group, there were the most extensive new bone tissue and the least fibrous tissue among the four groups. In the eighth week, a lot of new bone tissue but still lots of fibrous tissue were observed in the DM + HP66 group, whereas the fusion of tabular bone was observed in the DM + BHP66, DM + VHP66, and DM + BVHP66 groups (Figure 10A). Moreover, only in the DM + BVHP66 group, the tabular bone fused, bridging the fracture in the eighth week (Figure 10A).

Masson’s trichrome staining results were consistent with H&E staining results, which proved that the new bone tissue in the DM + HP66 group was the least and most immature compared with that in other groups in the fourth and eighth weeks. In all groups, new bone tissue increased over time and gradually replaced fibrous tissue. The most extensive and mature new bone tissue was observed in the DM + BVHP66 group in the eighth week (Figure 10B).

Similarly, staining for CD31 was performed to identify capillaries in the eighth week, which demonstrated that new capillary formation was less in the DM + HP66 and DM + BHP66 groups than in the DM + VHP66 and DM + BVHP66 groups (*P* < 0.05, Figure 10C). Furthermore, in the DM + BVHP66 group, the most substantial angiogenesis was observed in all groups (*P* < 0.05, Figure 10C).

## Discussion

Among the well-known consequences of DM, its impact on poor fracture healing has drawn increasing attention (Henderson et al., 2019). In this study, we constructed and proposed a dual peptide sustained-release system based on n-HA/PA66 scaffold to enhance diabetic fracture healing. Firstly, the peptide successfully anchored on the scaffold’s surface through covalently bonding and realized a sustained-delivery mode for 14 days. Secondly, the experimental results indicated that the BMP-2 peptide, combined with the VEGF peptide, could collaboratively improve osteogenesis and angiogenesis in diabetic bone regeneration. Thirdly, the results showed that fractures in both the non-DM group and the DM + BVHP66 group reached a complete healing in the eighth week, strongly proving that the BVHP66 scaffold can be a promising choice for rescuing the deleterious effect of DM on fracture healing.

The *in vivo* experiments implied that fracture healing was significantly worse and slower and bone formation was less in the DM group than in the non-DM group (Figures 9A,B). One primary reason for this is that stem cells, which act as the critical factor for bone regeneration, are deteriorated in HG condition (Sun et al., 2020; Xiang et al., 2020). As our *in vitro* results depicted, the proliferation and osteogenic differentiation abilities of rBMSCs were worse in the HG + HP66 group than in the CON + HP66 group (Figures 5C, 6, 7). Kim and Schafer (2016) reported that BMSCs switched the fate of differentiation from osteoblasts toward adipose cells in HG environment, increasing the amount of fat tissue in the fracture callus, thus hampering the bone healing process. Additionally, as exhibited in Figure 8A, HG condition contributed to cell viability of HUVECs in the short term but inhibited cell growth in the long term. The tube formation ability of HUVECs was worse in the HG + HP66 group than in the CON + HP66 group (Figures 8B–H). Likewise, Mangialardi et al. (2019) pointed that diabetes caused capillary rarefaction and compression of arteriole size in bone marrow, destabilizing the integrity of the microvasculature. Previous studies confirmed that the alterations in the extracellular presence of bone regeneration-related factors within diabetic tissues might inhibit the proliferation and function of stem cells (Qiao et al., 2018; Yu et al., 2019). In this work (Figures 7A,B), BMP-2 and VEGF genes of rBMSCs exhibited lower expression levels in HG condition than in standard glucose condition, consistent with a previous report (Hu and Olsen, 2016). It is a remarkable fact that bone regeneration is a time-bound process; thus, the presence of bone regeneration-related molecules is essential in the early stage (Mizuno et al., 2000). Thus, supplying these two bioactive molecules in advance is an effective strategy to overcome the obstruction of diabetic fracture healing. In detail, VEGF can regulate vascular development and promote endochondral and intramembranous ossification (Hu and Olsen, 2016). BMP-2 plays a role in enhancing the osteoid matrix’s secretion, which mineralizes to form mature bone tissue (Salazar et al., 2016). BMP-2 and VEGF growth factors have been used in regenerative medicine, whereas a series of disadvantages, such as high immunogenicity, limits their further application (James et al., 2016). Alternatively, it was reported that, compared with growth factor, peptide showed almost the same biological activity as specific amino acid sequence with fewer side effects because of its small size (Maia et al., 2013). Then, we need an appropriate release carrier to load peptide so that peptide could directly stimulate cell activity around the fracture site.

The n-HA/PA66 scaffold is a preferable choice. As some papers reported, this scaffold exhibited a very close composition and structure to natural bone tissue (Wang et al., 2002). Moreover, its porous structure with suitable pore size and porosity could provide a 3D environment for cell attachment and proliferation, thus promoting tissue ingrowth (Wang et al., 2007). A highly porous structure decreases the mechanical strength, whereas the n-HA/PA66 scaffold reaches a balance between porosity and mechanical strength required by bone tissue engineering (Wang et al., 2007). Moreover, the mechanical strength of the shaped n-HA/PA66 scaffold was comparative to that of natural bone, contributing to the reconstruction of even load-bearing bone (Hutmacher, 2000). Then, it is important to apply an appropriate method to bind peptide with this scaffold together. Conventionally, bioactive molecules are physically adsorbed on the scaffold, causing them to be rapidly released into neighboring tissue. However, this rapid release mode cannot satisfy the need of continuous bioactive molecule delivery to stabilize new formed tissue. Hence, it is superior to apply a method that can provide a sustained-release model and ensure local retention of required bioactive molecules. Alternatively, it was reported that bioactive molecules could be covalently bonded to the scaffold’s surface to achieve a stable integration and obtain a sustained-release behavior (Mohammadi et al., 2018). As the n-HA/PA66 scaffold contains free carboxyl groups, which can interact with the peptides’ amino groups to form covalent bonds, we further confirm that the n-HA/PA66 scaffold is a preferable release carrier. In this work, the BMP-2 and VEGF peptides were successfully anchored on the surface of n-HA/PA66 scaffold employing covalent bonds (Figures 3B,C). The peptide release experiments *in vitro* suggested that the cumulative release profiles of BMP-2 and VEGF peptides were similar, which held the properties of the initial burst-release and the subsequent slow-sustained release (Figure 3D). The initial burst-release for the BVHP66 scaffold could be relative to the release of peptide physically adsorbed on this porous scaffold, whereas its subsequent slow-sustained release may be ascribed to the hydrolysis rates of covalent bonds (Lin et al., 2010). In the beginning, the rapid release could supply the BMP-2 and VEGF peptides timely, and the followed slow-sustained release could contribute to regulating cell activity continuously. The BVHP66 scaffold fits the specific need for diabetic bone regeneration; it delivers BMP-2 and VEGF peptides that are lacking in diabetic condition to reach their cell targets, and the release kinetics of BMP-2 and VEGF peptides are desirable for related stem cells in diabetic condition to mimic the physiological process of normal fracture healing.

Bone regeneration involves complex physiological processes that are generally mediated by multiple bioactive molecules. The release of two types of bioactive molecules, the so-called dual release system, is promising for bone regeneration (Kim and Tabata, 2015). Some studies have reported that bone regeneration can be significantly improved by the combined BMP-2 and VEGF growth factors. Dashtimoghadam et al. (2020) demonstrated conjugation of BMP-2 growth factor onto monodisperse polymeric microcarriers encapsulating VEGF growth factor, which displayed an additive effect on bone regeneration. Moreover, in the study of Godoy-Gallardo et al. (2020), BMP-2 was bound onto the inner polydopamine layer, whereas VEGF was immobilized onto the outer one, and the two growth factors played a synergistic effect on bone regeneration. Compared with a fast–slow VEGF growth factor delivery followed by a slow-sustained release of BMP-2 growth factor for normal bone in these two studies, we provide a favorable option that supplies BMP-2 and VEGF molecular signals in the form of peptides with greater advantage, and a burst-release followed by slow-sustained release mode of BMP-2 and VEGF peptides is better to fit the specific need of diabetic bone regeneration. Moreover, the effect of the combination of BMP-2 and VEGF peptides with this proper release mode on diabetic bone regeneration has not been evaluated yet. As a result, we aimed to evaluate the effect of the BVHP66 scaffold on osteogenesis and angiogenesis in the diabetic environment. Based on our experiments, rBMSCs could successfully attach to the surface of BVHP66 scaffold (Figures 5A,B), and the number of rBMSCs seeded on the BVHP66 scaffold was increased over time (Figure 5C). As delineated in Figure 5C, the sustained delivery of the combined BMP-2 and VEGF peptides by the n-HA/PA66 scaffold can synergistically enhance cell proliferation of rBMSCs and attenuate the negative effect of HG on proliferation of rBMSCs. Moreover, it was demonstrated that the dually sustained-release of BMP-2 and VEGF peptides by the BVHP66 scaffold could synergistically enhance osteogenic differentiation and mineralization of rBMSCs, play a role in both the early and late stages, and rescue the inhibitory effect of HG condition on rBMSCs (Figures 6, 7). On the other hand, the BVHP66 scaffold and VHP66 scaffold showed no evident difference in abilities of promoting the proliferation and tube formation of HUVECs under HG conditions, whereas the BHP66 scaffold exhibited no significant effects as above, indicating that the sustained-release of BMP-2 and VEGF peptides did not exhibit a cooperative effect (Figure 8).

To evaluate the effectiveness of the BVHP66 scaffold on bone regeneration, the fracture model of diabetic rats was utilized. An STZ-induced diabetic rat is a typical animal model of T1DM, and blood glucose mimics the metabolic characteristic of DM in humans (Wu and Huan, 2008). Observations based on X-ray, micro-CT (Figure 9), and histological experiments (Figures 10A,B) illustrated that bone formation was considerably accelerated by the BVHP66 scaffold and the dual delivery of BMP-2 and VEGF peptides had an additive effect. Additionally, the IHC results (Figure 10C) indicated that the BVHP66 scaffold could more strongly enhance angiogenesis in the diabetic rat model than the BHP66 scaffold and the VHP66 scaffold. Moreover, the BHP66 scaffold exhibited no more significant angiogenic effect than the HP66 scaffold. The above results indicated that the angiogenic effect of the combined BMP-2 and VEGF peptides *in vivo* varied from that *in vitro*. The two peptides did not display mutual enhancement in the *in vitro* experiments, whereas their mutual synergistic effect was notable in the *in vivo* experiments. That is because the increased angiogenesis can bring BMSCs, oxygen, nutrition, and minerals necessary for mineralization *in vivo*, whereas such physiological microenvironment is hard to mimic *in vitro*. Moreover, osteogenic factors, released from blood vessels, promote the differentiation and mineralization of osteoblast (Matsubara et al., 2012). In turn, maturing osteoblasts generate angiogenesis-related molecules to support further angiogenesis (Hu and Olsen, 2016). Bone is a highly vascularized tissue in which blood vessels and bone cells interact with each other, and VEGF stimulates the formation of supportive vascular networks of HUVECs, which enhance the effect of BMP-2 on bone-forming of BMSCs (Hankenson et al., 2015). This once again demonstrated the linkage between osteogenesis and angiogenesis in bone regeneration.

Overall, the BVHP66 scaffold provides a promising therapeutic option that satisfies the specific need of diabetic bone regeneration and synergistically promotes osteogenesis and angiogenesis.

## Conclusion

In conclusion, DM impairs osteogenesis and angiogenesis and delays fracture healing. To address this issue, we have designed and synthesized a bioactive scaffold BVHP66 that can achieve BMP-2 and VEGF peptides sustained-release for promoting diabetic fracture healing efficiently. In *in vitro* experiments, the sustained-release of BMP-2 and VEGF peptides could synergistically enhance rBMSCs proliferation and osteogenic differentiation, and the VEGF peptide also could contribute to proliferation and tube formation of HUVECs in HG condition. Furthermore, in *in vivo* experiments, the prepared BVHP66 scaffold synergistically promoted osteogenesis and angiogenesis, leading to the complete fracture healing of the fractured diabetic rats in the eighth week. These suggest a promising role of the novel developed BVHP66 scaffold for attenuating the inhibitory effect of DM on fracture healing.

## Data Availability Statement

The original contributions presented in the study are included in the article/supplementary material, further inquiries can be directed to the corresponding author/s.

## Ethics Statement

The animal study was reviewed and approved by the Ethical Committee of Harbin Medical University (approval number, SYDW2019-2).

## Author Contributions

JL carried out the experiments and drafted the manuscript. JW and AL analyzed the data. AL, HL, and JS helped with the surgery. HQ reviewed the data and revised the manuscript. All authors read and approved the final manuscript.

## Conflict of Interest

The authors declare that the research was conducted in the absence of any commercial or financial relationships that could be construed as a potential conflict of interest.
